# Genome-Wide Association Mapping to Identify Genetic Loci for Cold Tolerance and Cold Recovery During Germination in Rice

**DOI:** 10.3389/fgene.2020.00022

**Published:** 2020-02-21

**Authors:** Ranjita Thapa, Rodante E. Tabien, Michael J. Thomson, Endang M. Septiningsih

**Affiliations:** ^1^ Department of Soil and Crop Sciences, Texas A&M University, College Station, TX, United States; ^2^ Texas A&M AgriLife Research Center, Beaumont, TX, United States

**Keywords:** low temperature stress, cold tolerance, cold recovery, germination, genome-wide association study, SNP, QTL, rice

## Abstract

Low temperature significantly affects rice growth and yield. Temperatures lower than 15°C are generally detrimental for germination and uniform seedling stand. To investigate the genetic architecture underlying cold tolerance during germination in rice, we conducted a genome-wide association study using a novel diversity panel of 257 rice accessions from around the world and the 7K SNP marker array. Phenotyping was conducted in controlled growth chambers under dark conditions at 13°C. The rice accessions were measured for low-temperature germinability, germination index, coleoptile length under cold stress, plumule length at 4-day recovery, and plumule length recovery rate. A total of 51 QTLs were identified at *p* < 0.001 and 17 QTLs were identified using an FDR < 0.05 across the different chilling indices with the whole panel of accessions. At the threshold of *p* < 0.001, a total of 20 QTLs were identified in the subset of *japonica* accessions, while 9 QTLs were identified in the subset of *indica* accessions. Considering the recurring SNPs and linked SNPs across different chilling indices, we identified 31 distinct QTL regions in the whole panel, 13 QTL regions in the *japonica* subset, and 7 distinct QTL regions in the *indica* subset. Among these QTL regions, three regions were common between the whole panel and *japonica*, three regions were common between the whole panel and *indica*, and one region was common between *indica* and *japonica*. A subset of QTL regions was potentially colocalized with previously identified genes and QTLs, including 10 from the *japonica* subset, 4 from the *indica* subset, and 6 from the whole panel. On the other hand, a total of 21 potentially novel QTL regions from the whole panel, 10 from the *japonica* subset, and 1 from the *indica* subset were identified. The results of our study provide useful information on the genetic architecture underlying cold tolerance during germination in rice, which in turn can be used for further molecular study and crop improvement for low-temperature stressed environments.

## Introduction

Rice is more susceptible to cold stress than other cereal crops due to its origin in the tropical and subtropical regions (Zhao et al., 2017). Low temperature causes major stress for rice growing in 25 countries (Cruz et al., 2013) and to more than 15 million ha of rice grown worldwide (Bai et al., 2016). One of the major challenges for rice production under direct-seeded cultivation, especially in high altitude regions in the tropics or regions with temperate climates, is low-temperature sensitivity during the germination stage (Schläppi et al., 2017). Cold stress during germination causes poor germination and retarded plant growth. Vigorous germinated seedling is a necessity for good plant establishment. Breeding of rice cultivars with tolerance of low temperature, however, has been challenging due to various factors: response of rice plants to cold varies with growth stages (Liu et al., 2015); low-temperature tolerance is controlled by quantitative loci where many genes with small effects contributing to the phenotype (Ji et al., 2009); and epistatic interaction among alleles at unlinked loci (Zhang et al., 2014a). A wide range of variations to cold tolerance among *Oryza sativa* has been reported where accessions of the *japonica* subspecies were generally being more tolerant than *indica* (Baruah et al., 2009). A few studies have been performed to improve cold tolerance of the *indica* cultivars using *japonica* cultivars; however, due to lack of genetic diversity in *japonica* germplasm, further improvement of *japonica* cultivars has been quite challenging (Zhang et al., 2014a).

Thus far, only very few genes controlling chilling tolerance have been identified in different stages of rice growth (Cruz et al., 2013; Zhang et al., 2014b; Zhang et al., 2014c). The first gene reported for low-temperature germinability was *qLTG3-1*, where the gene encodes for a secreted hybrid glycine-rich protein and a single nucleotide was the causal polymorphism (Fujino et al., 2008). It is highly imperative to identify additional chilling tolerance QTLs and genes to better understand the mechanisms of chilling tolerance in rice and to assist in developing high-yielding rice with higher tolerance of cold during germination. QTL mapping and genome-wide association study (GWAS) are two widely used tools to discover the genetic control of complex traits. Most of the published data on genetic loci controlling chilling tolerance in rice were obtained by bi-parental mapping populations from *O. sativa* ssp *indica* X *O sativa* ssp *japonica* crosses where *japonica* subspecies usually used as the donors for cold tolerance (Mackill and Lei, 1997; Cruz and Milach, 2004; Mao and Chen, 2012; Ma et al., 2015). The major drawback of bi-parental mapping is the limitation of genetic background to parental lines. More recently, GWAS has also been utilized to study cold tolerance in rice, with the advantage of scanning a large number of accessions for genetic loci controlling this trait. These studies have led to the discovery of QTLs associated with low-temperature germination during seedling stage and plumule growth recovery after chilling stress in rice. Pan et al. (2015) identified 22 QTLs for cold tolerance during germination stage using SSR markers in 174 Chinese accessions. Sales et al. (2017) detected 24 SNPs associated with low-temperature germination and growth rate at low temperature; while Shakiba et al. (2017) reported 42 QTLs controlling cold tolerance at seedling stage. Fujino et al. (2015) conducted GWAS mapping with 117 markers using a Hokkaido rice core panel, comprising 63 Japanese landraces and breeding lines and discovered 6 QTLs for cold tolerance at heading stage and 17 QTLs for low-temperature germinability. Lv et al. (2016) reported 132 loci associated with 16 traits evaluated under natural chilling and cold shock stress using a large collection of 529 rice accessions with more than 4.35 million SNP markers. Schläppi et al. (2017) identified a total of 48 QTLs for chilling tolerance in 202 *O. sativa* accessions from the USDA mini-core collection.

Various methods, traits measured, and temperatures have been used to identify underlying genes of cold tolerance in rice during germination stage. Shakiba et al. (2017) evaluated cold tolerance at germination stage using the “ragdoll method” exposed at 12°C for 35 days. Sales et al. (2017) germinated rice seeds for 21 days at 15°C to evaluate the cold tolerance during germination stage. Schläppi et al. (2017) conducted GWAS during germination, seedling and recovery stage in 202 *O. sativa* accessions. For germination cold tolerance, growth rate of plumule after 30 days of cold exposure at 10°C was measured 4 days after recovery at 28 ± 1°C. The mean length of 2-week old seedlings at V2 stage was recorded before cold exposure and again after 1 week of recovery, the length of the recovered seedlings was measured. The growth at 28 ± 1°C following a 1-week chilling stress treatment at 10 ± 1°C was recorded to estimate leaf recovery growth rate after cold exposure. These different stress treatments and different indicators used to study cold tolerance have resulted in variation in the number and location of the identified QTLs (Zhang et al., 2014c). Cruz and Milach (2004), however, suggested that variation in coleoptile growth and percentage of seeds superior to 5-mm coleoptile length at cold temperatures were sufficient to identify cold-tolerant genotypes. The differences in seedling vigor among genotypes may cause difficulty in the identification of cold-tolerant lines. Because of this, several researchers have emphasized the evaluation of test entries in both control (ambient) and cold temperature to enable the separation of seedling vigor from cold tolerance (Sales et al., 2017).

In this study, we performed GWAS on a novel rice diversity panel of 257 accessions using a 7K rice SNP array (S. McCouch, M. Thomson, and K. Morales, personal communication). The objectives of this study were to evaluate the diversity rice panel for cold tolerance and cold recovery during the germination stage and to identify QTLs and the underlying candidate genes.

## Materials and Methods

### Rice Accessions

The 257 rice accessions/lines used in this study were obtained from the USDA-ARS National Small Grains Collection (Aberdeen, Idaho), the Genetic Stocks-Oryza (GSOR) collection located at the USDA-ARS Dale Bumpers National Rice Research Center (USDA-ARS DBNRRC; Stuttgart, AR), and the inbred rice breeding program at the Texas A&M AgriLife Research Center in Beaumont, Texas (**Supplementary Table 1**). This panel represented accessions or breeding lines belonging to the *indica* subspecies (*indica* and *aus)*, the *japonica* subspecies (*aromatic*, *tropical japonica*, and *temperate japonica*), *O. glaberrima*, and several Nerica lines (derivatives of *O. sativa*/*O. glaberrima* interspecific crosses). This novel diversity panel was selected to represent geographic diversity, including 62 accessions from South Asia, 50 from Central and Western Asia, 27 from Southeast Asia, 8 from East Asia, 34 from Africa, 15 from Europe and Russia, 6 from Latin America, and 55 from North America. Seed multiplication was performed in the Texas A&M AgriLife Research Center in Beaumont, TX (summer 2016). Seeds from one panicle of each accession were direct-seeded in a single row for seed multiplication in summer 2017. All plants were maintained following the Texas production guidelines. After maturity, per plant harvest was performed and the seeds were dried in a heated air dryer at 37°C for 5 days and then stored at 4°C. To break the dormancy, seeds were incubated at 50°C for 5 days. The germination test of each accession was performed using the roll paper method (http://www.knowledgebank.irri.org/step-by-step-production/pre-planting/seed-quality).

### Indices for Evaluating Cold Tolerance

To screen for the cold tolerance variability in the collected germplasm, different parameters were used, including low-temperature germinability (LTG), germination index (GI), coleoptile length under cold stress (CLC), plumule length at 4-day recovery (PLR), and plumule length recovery rate (PLRR) that are described in detail below. The experiment was conducted in a growth chamber in a controlled-dark condition following a completely randomized design with three replications, and 30–40 seeds per replication were used. Seeds of all accessions were rinsed with 5% Tween-20 for 5 min followed by thorough rinsing with 10% bleach (sodium hypochlorite) for 10 min and washed with autoclaved distilled water 3 times to prevent contamination.

For control samples, 30–40 sterilized seeds were placed on water-soaked filter paper placed in the petri dishes. The petri dishes were then wrapped in aluminum foil and placed for germination in a growth chamber maintained at 30°C. The experiment was conducted in a completely randomized design and the dark condition was provided to mimic the natural dark condition under the soil during the germination stage. After 7 days of germination, the average germination percentage per accession was taken from all the three replicates.

#### Low-Temperature Germinability (LTG) and Germination Index (GI)

Surface sterilized seeds were incubated in water-soaked filter paper in petri dishes, 30–40 seeds were placed in each petri dish and these were then wrapped with aluminum foil. For each entry, three plates were randomly distributed in the growth chamber set at 13°C temperature. Another set was grown in the growth chamber at 30°C temperature as controls. Germinated seeds were counted in each petri dish obtained after 7 days in the 30°C growth chamber (control) and after 28 days in the 13°C growth chamber (cold treatment). Germination was defined as visible coleoptile emergence (>5 mm) through the hull. The low-temperature germinability (LTG) was calculated as the percent of seeds germination at 13**°**C after 28 days. The mean LTG scores were recorded from three petri dishes and normalized with the mean percent germinability of seeds at 30°C (NTG) which was used to calculate the germination index (GI). GI was determined as LTG divided by NTG times 100.

#### Coleoptile Length Under Cold Stress (CLC)

After counting the germinated seeds, all the germinating seedlings were placed on a sterile black background paper along with the ruler for photographs. The images of all the germinating seeds from each replication were then taken with a Pentax camera. Later, the images were imported to ImageJ software and the coleoptile length of all the germinated seeds was measured and averaged to represent the mean of coleoptile length of each accession after cold exposure. The arithmetic means of the measurement were used for GWAS mapping.

#### Plumule Length At 4-Day Recovery (PLR) and Plumule Length Recovery Rate (PLRR)

After photographing all germinating seeds, the seedlings were returned to their corresponding petri dish, covered with foil and then were moved to a growth chamber maintained at 30**°**C and were kept for 4 days. After the recovery period of 4 days, pictures were taken, and plumule lengths were measured using ImageJ. The average from three replication was taken as plumule length after recovery (PLR) for each accession. The mean plumule growth rate after cold germination was estimated by subtracting the mean coleoptile length after 28 days at 13**°**C from the mean plumule length on day 4 at 30**°**C after recovery and dividing the obtained value by 4 to represent plumule length recovery rate (PLRR). The PLRR value, therefore, indicates the growth rate of the plumule over a period of 4 days under normal conditions (30**°**C).

### Genotyping

The young leaves were collected from the field in Beaumont, Texas in 2017 and sent to a genotyping service lab for DNA extraction and genotyping (Eurofins BioDiagnostics, Inc., River Falls, WI). Genotyping of all the accessions was performed using a 7K Illumina iSelect custom-designed array by following the Infinium HD Array Ultra Protocol. The 7K array, called the C7AIR, was designed by the McCouch Lab at Cornell University and consists of 7,098 SNPs. The Cornell_7K_Array_Infinium_Rice (C7AIR) design represents an improved version of Cornell_6K_Array_Infinium_Rice (C6AIR) (Thomson et al., 2017). Genotype data used in this study were called using Genome Studio software (Illumina, USA). SNPs of call rate <90% and minor allele frequency <5% were removed from the dataset. The quality of each SNP was confirmed manually by re-clustering. For our study, a subset of 5,185 high-quality SNP markers obtained after removal of rare allele markers at 5% or less and heterozygosity of more than 20% were used to perform the genome-wide association analysis.

### General Statistics, Population Structure, and Association Mapping

The basic statistics for all traits were analyzed, including heritability (Singh et al., 1993). Spearman's correlation coefficients between the chilling indices were also calculated using R software version 3.5.1 (Lenth, 2016). Additionally, the mean and standard error for the five selected traits were calculated for each sub-population generated from the STRUCTURE program (Pritchard et al., 2000); comparisons were then made between these distributions to the generally more tolerant *temperate japonica* population using a Student's *t*-test.

The Bayesian model of the Markov Chain Monte Carlo (MCMC) implemented in the STRUCTURE program (Pritchard et al., 2000) was used to estimate the population structure. The burn-in length and number of replications were both set to be 100,000. For each number of populations (Q), five iterations were performed for the number of populations 2 to 10. The Structure Harvester program (Earl, 2012) was used to perform the analysis. The coefficient of ancestry (Q) threshold was defined at 70% to refer an individual with its inferred ancestry from one single group; while the accessions which were unable to be assigned to only one group were determined as mixed ancestry. We also used the Bayesian clustering program fastStructure (Raj et al., 2014) to estimate the different levels of Q (Q = 1–10).

GWAS of all *japonica* (*temperate japonica*, *tropical japonic*a, *aromatic*), *indica* (*aus* and *indica*), and the whole panel were conducted using their corresponding data sets. The Genome Association and Prediction Integrated Tool (GAPIT) package (Lipka et al., 2012) with a genotype matrix of 5185 SNPs and a phenotype matrix of 257 accessions were used to perform the GWAS analysis. To predict the genomic regions associated with the traits, we used mixed linear model (MLM) of GAPIT (Zhang et al., 2010). For MLM, we used both kinship (K) matrix as the variance-covariance matrix between the individuals and population structure (Q) matrix to control false positive. The structure data was obtained from the STRUCTURE software (Pritchard et al., 2000) and the kinship relationship matrix (K) was obtained from the TASSEL 4.0 software (Bradbury et al., 2007). For association mapping in *japonica* and *indica* sub-populations, considering the low sample size, the MLM model of GAPIT using principal components (PCs) was used to avoid overcorrection (Hsu and Tung, 2015).

The MLM model used is: *Y*  =  *βX* + *γP* + *Zu* + *ϵ*; where *Y* is the vector of the phenotypic data, *X* is the vector of genotypic data, *β* represents the SNP effect, *P* is the vector of the Q matrix representing population structure, *γ* is the effect of population structure, *u* refers to the random effect from kinship, Z is the Kinship matrix, and *ϵ* corresponds to random error. The expected *p*-values versus the observed *p*-values test statistics for the SNP markers were plotted (QQ plot) to assess the control of type I errors under multiple run parameters. The markers were defined to be significantly associated to chilling indices based on *p* < 0.001. The extent of LD in rice on average ranges from 100 to 500 kb (Garris et al., 2005; Myles et al., 2009; Tung et al., 2010). Hence, we defined two or more SNPs positioned within ~250 kb as a single QTL. The Manhattan plot distribution chart was obtained by the R software. The percent variance explained by all significant SNPs discovered for each trait was estimated by subtracting the R^2^ of the model without SNP from R^2^ of the model with SNP (Zhang et al., 2010). Candidate genes at or near the QTLs identified in this study (within ~250 kb) were from the QTARO database (http://qtaro.abr.affrc.go.jp/; Yonemaru et al., 2010) and other previously published literature.

## Results

### Phenotypic Performance and Correlation Among Traits

Most of the rice accessions used in this study have more than 90% germination rate at 30**°**C. However, we observed a wide variation in coleoptile length (**Table 1**). In most cases, germination and coleoptile length were significantly decreased when the rice seeds were germinated at a low temperature of 13**°**C. Under cold exposure, LTG ranged from 0% to 100%. Cold temperature delayed the germination rate of rice seeds and many of the lines started germinating after 7 days of sowing. The range of the coleoptile length was found to be 0 cm to 1.69 cm; while the mean was 0.69 cm. The PLR and the PLRR ranged from 0 cm to 5.33 cm and 0 cm/d to 1.08 cm/d, with the mean values of 2.96 cm and 0.57 cm/day, respectively. Overall, the broad sense heritability estimations for all traits were high, ranging from 86.8% to 94.0% (**Table 1**).

**Table 1:** Descriptive statistics of each trait measured in the whole diversity panel.

Trait	Description	Measurement unit	Mean	Range	SE	H^2^
LTG	Low-temperature germinability	%	69.21	0–100	1.69	94.0
GI	Germination index	NA	72.47	0–107.60	1.76	87.7
CLC	Coleoptile length under cold conditions	cm	0.69	0–1.69	0.02	89.2
PLR	Plumule length after recovery	cm	2.96	0–5.33	0.07	90.8
PLRR	Plumule length recovery rate	cm/d	0.57	0–1.08	0.01	86.8

**Figure 1:**
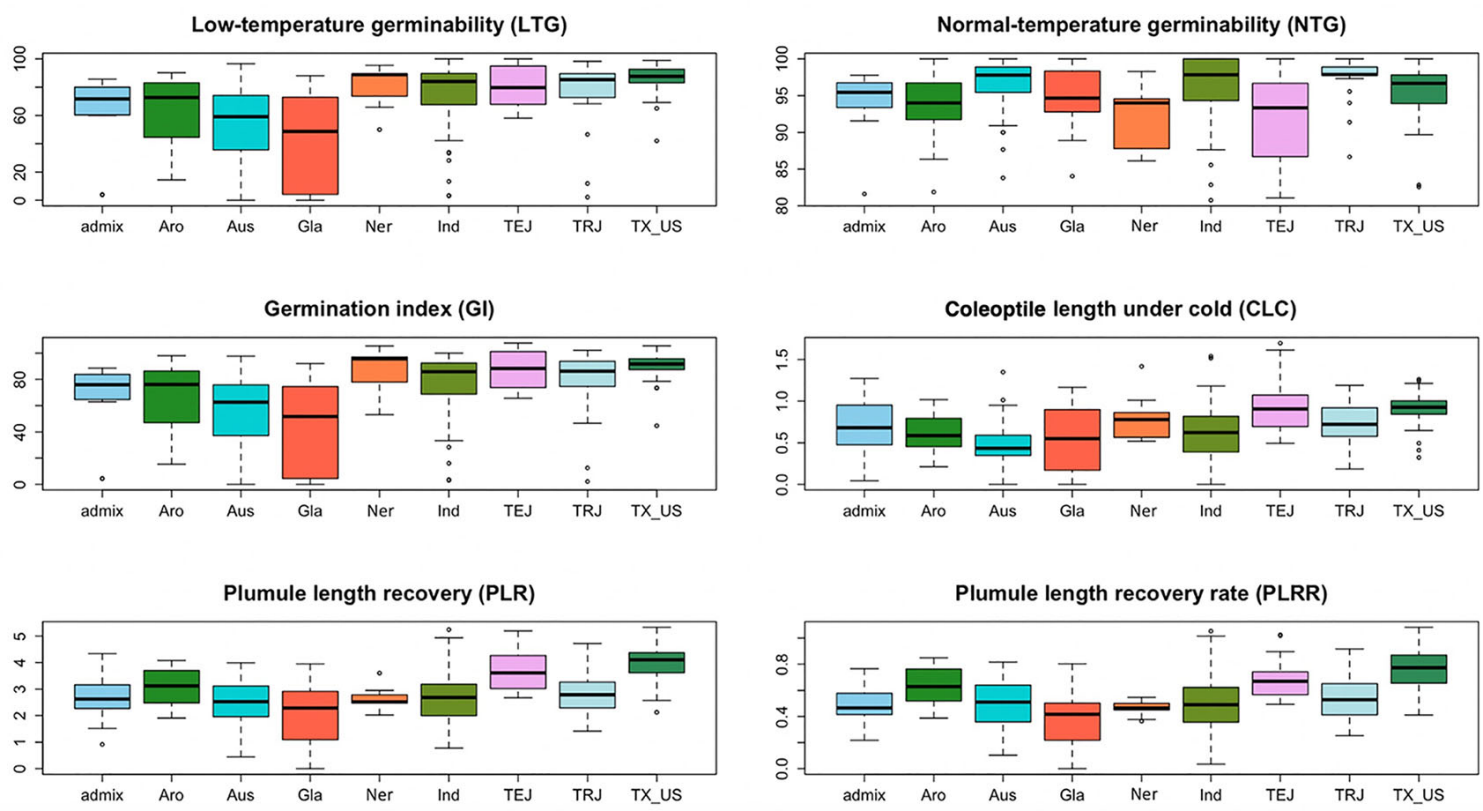
The box plots from all sub-populations identified by the STRUCTURE software presented for all the traits measured: low-temperature germinability (LTG), percentage germination under normal condition (NTG), germination index (GI), coleoptile length under cold (CLC), plumule length recovery (PLR), and plumule length recovery rate (PLRR).

Based on the population structure, the whole panel was categorized into nine sub-populations, including the admixtures (**Table 2**). We observed that among the highest LTGs were the group of Texas breeding lines and the US released varieties, followed by NERICA lines and *temperate japonica*, with the means of 86.2%, 80.81%, and 80.3%, respectively; while the lowest was *O. glaberrima* with the mean of 41.12%. Among the *japonica*, *aromatic* has the lowest LTG (64.17%), a similar rate to the *aus* group (53.76%). Interestingly, the *indica* lines used in our study (73.67%) had comparable germination rates under cold stress with several of the *japonica* lines. The three groups having the highest LTG are almost the same as CLC, with *temperate japonica* having the highest mean for CLC (0.94 cm), followed by Texas and US lines (0.91 cm) and then the NERICA lines (0.80 cm). Similarly, the smallest length for CLC was also observed in *aus* followed by *O. glaberrima* and *aromatic*. CLC of the *indica* (0.63 cm) was generally shorter compared to the *japonica* groups except for the *aromatic* (0.61 cm). The recovery process from cold stress was also evaluated using the PLR and PLRR parameters. For PLR, the Texas lines and *temperate japonica* had the longest plumule growth with the mean values of 3.96 cm and 3.68 cm, respectively; whereas the shortest growth was seen in *O. glaberrima* with a mean value of 2.07 cm. A similar trend was observed for PLRR (**Table 2**). The values of LTG and PLR were significantly lower for *O. glaberrima*, *aus*, and *admixtures* (**Table 2**; **Figure 1**). For CLC and PLRR, the values were significantly lower for *O. glaberrima*, *aus*, *aromatic*, *indica*, and admixtures. We also observed significantly higher PLRR of Texas lines compared to *temperate japonica* (*p* < 0.05); whereas no significant difference of CLC was observed between Texas lines and *temperate japonica*. Very high significant positive correlations between all the chilling indices were detected (**Table 3**), albeit with different levels of significance ranging from 0.44 (between CLC and PLRR) to 0.97 (between LTG and GI). The results showed that rice accessions having good germination under cold stress in general also having higher coleoptile length under cold stress and high recovery rate as well.

**Table 2:** Phenotypic performance of nine sub-populations generated within the whole diversity panel.

Sub-pop^a^	# samples	LTG^b^ (%)	NTG^c^ (%)	GI^d^	CLC^e^ (cm)	PLR^f^ (cm)	PLRR^g^ (cm/d)
Admixture	11	60.73 ± 8.85	94.08 ± 4.60	63.78 ± 9.18*	0.67 ± 0.10*	2.63 ± 0.27**	0.49 ± 0.04**
Aromatic	20	64.17 ± 5.26*	93.59 ± 1	68.43 ± 5.50**	0.61 ± 0.05***	3.13 ± 0.16*	0.63 ± 0.03
Aus	53	53.76 ± 3.86***	96.74 ± 0.48***	55.25 ± 3.93***	0.48 ± 0.03***	2.46 ± 0.11***	0.5 ± 0.02***
*Oryza glaberrima*	20	41.12 ± 7.85***	94.74 ± 0.92	42.47 ± 8.07***	0.54 ± 0.09***	2.07 ± 0.27***	0.38 ± 0.05***
*Nerica*	9	80.81 ± 4.98	92.48 ± 1.45	87.74 ± 5.85	0.8 ± 0.09	2.65 ± 0.15***	0.46 ± 0.02***
*Indica*	48	73.67 ± 3.56	96.42 ± 0.64***	76.34 ± 3.65*	0.63 ± 0.05***	2.67 ± 0.15***	0.51 ± 0.03***
*Temperate japonica*	30	80.3 ± 2.59	92.17 ± 0.94	87.04 ± 2.56	0.94 ± 0.06	3.68 ± 0.14	0.69 ± 0.03
Texas	44	86.2 ± 5.43	95.51 ± 0.67**	90.21 ± 5.60	0.91 ± 0.06	3.96 ± 0.17	0.76 ± 0.04*
*Tropical japonica*	22	77.16 ± 5.43	97.4 ± 0.67***	79.24 ± 5.60	0.74 ± 0.06*	2.89 ± 0.16***	0.54 ± 0.04**

a These sub-populations generated by the STRUCTURE software.

b Low-temperature germinability.

c Normal-temperature germinability.

d Germination index.

e Coleoptile length after cold exposure.

f Plumule length after recovery.

g Plumule length recovery rate.

*p-value < 0.05; **p-value < 0.01; ***p-value < 0.001.

**Table 3:** Correlation analysis of different cold tolerance traits among all accessions.

Trait	LTG^a^	GI^b^	CLC^c^	PLR^d^	PLRR^e^
**LTG**	1	0.97***	0.64***	0.58***	0.47***
**GI**	0.97***	1	0.65***	0.60***	0.50***
**CLC**	0.64***	0.65***	1	0.67***	0.44***
**PLR**	0.58***	0.60***	0.67***	1	0.96***
**PLRR**	0.47***	0.50***	0.44***	0.96***	1

a Low-temperature germinability.

b Germination index.

c Coleoptile length after cold exposure.

d Plumule length after recovery.

e Plumule length recovery rate.

***p-value < 0.0001.

### GWAS for Identification of QTLs

The population structure analysis for the whole accessions identified nine sub-populations. The results for the *japonica* and *indica* group-specific GWAS, however, showed an overcorrection for the population structure when both population structure (Q) and kinship matrix (K) were considered in the mixed model (*japonica* MLM and *indica* MLM). To avoid this overcorrection and to control the false-negative results, a GAPIT model considering the principal components (PCs) were used to individually analyze the *indica* and *japonica* varietal groups. Only two main sub-populations were observed in the *indica* group and three sub-populations were observed in the *japonica* group as depicted by the PCA plot results from GAPIT output. In *indica* group, the first PC and second PC explained 28% and 5% of the total variance, respectively, whereas in *japonica* group, the first PC, second PC, and third PC explained 32%, 5%, and 4% of the total variance, respectively.

### GWAS of Chilling Tolerance Indices for All Accessions

**Figure 2:**
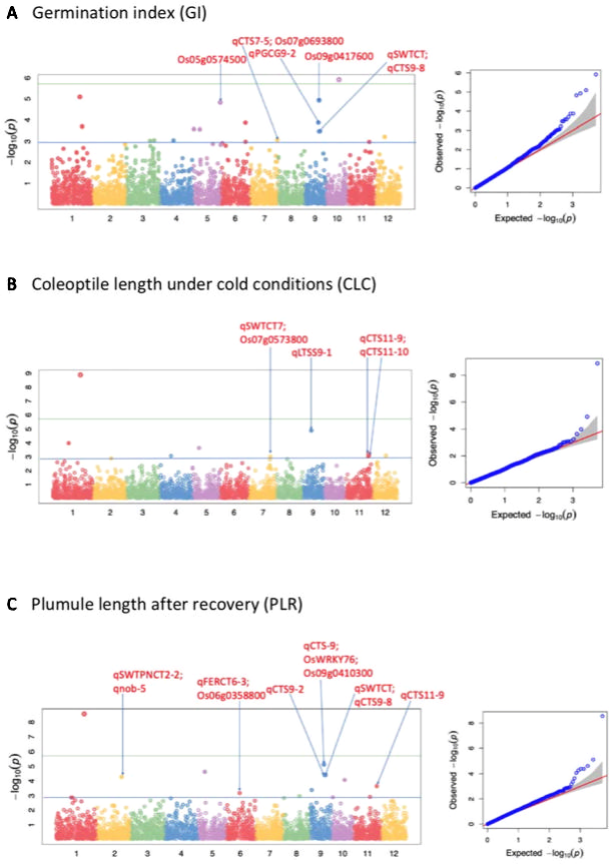
Selected Manhattan and QQ plots of the whole panel for germination index (GI), shoot length under cold (CLC), and plumule length recovery (PLR). The solid blue line shows the p-value 0.001 significant threshold; while the solid green line shows the Bonferroni correction.

A total of 51 QTLs were identified at *p* < 0.001, with 11, 15, 9, 9, and 7 QTLs were discovered to be significantly associated with LTG, GI, CLC, PLR, and PLRR, respectively (**Table 4**; **Supplementary Table 2**; **Figure 2**; **Supplementary Figure 1**). Out of the 51 QTLs, 17 of them were detected at FDR < 0.05, with 4, 4, 2, 6, and 1 QTLs were found to be associated with LTG, GI, CS, PLR, and PLRR, respectively (**Table 4**). The amount of phenotypic variance explained (R^2^) ranged from 0.5% to 20.6% for LTG, 1.3% to 4.8% for GI, 1.8% to 12.9% for CLC, 0.6% to 8.6% for PLR, and 0.8% to 10.0% for PLRR.

**Table 4:** QTLs with FDR < 0.05 detected in the whole panel, *japonica* and *indica* subsets, and colocalized genes and QTLs.

QTL ID	Trait^a^	Colocated QTL in this study	Group	Chr.	Position (bp)	*p*-value	FDR	R^2^	Potentially colocated QTL/gene	Reference
*qPLRR-1*	PLRR	*qLTG-1-1; qGI-1-1; qCLC-1-2; qPLR-1*	Full set	1	2,994,2776	4.39E−29	2.28E−25	10.0		
*qLTG-1-1*	LTG	*qGI-1-1; qCLC-1-2; qPLR-1; qPLRR-1*	Full set	1	2,9942,776	2.70E−14	1.40E−10	20.1		
*qCLC-1-2*	CLC	*qLTG-1-1; qGI-1-1; qPLR-1; qPLRR-1*	Full set	1	29,942,776	1.29E−09	6.69E−06	12.9		
*qPLR-1*	PLR	*qLTG-1-1; qGI-1-1; qCLC-1-2; qPLRR-1*	Full set	1	29,942,776	2.74E−09	1.42E−05	8.4		
*qGI-1-1*	GI	*qLTG-1-1; qCLC-1-2; qPLR-1; qPLRR-1*	Full set	1	29,942,776	8.02E−06	0.0191873	1.5		
*qPLR-2*	PLR	*qPLRR-2*	Full set	2	26,231,409	5.31E−05	0.0458891	1.4	*qSWTPNCT2-2; qnob-5*	Shakiba et al., 2017
*qGI-5-3*	GI		Full set	5	28,831,954	1.48E−05	0.0191873	3.1	*Os05g0574500*	Chen et al., 2011
*qLTG-5-1*	LTG	*qGI-5-1*	Full set	5	805,425	1.83E−05	0.0237779	3.7		
*qPLR-5*	PLR	*qLTG-5-2; qGI-5-2; qCLC-5*	Full set	5	7,195,992	2.35E−05	0.0405948	2.2		
*qGI-9-2*	GI		Full set	9	15,414,541	1.18E−05	0.0191873	4.8	*Os09g0417600*	Yokotani et al., 2013
*qPLR-9-2*	PLR	*qGI-9-1; qInPLR-9; qPLRR-9*	Full set	9	14,648,157	7.42E−06	0.0192423	3.2	*qCTS-9; OsWRKY76; Os09g0410300*	Peng et al., 2010
*qCLC-9*	CLC	*qJaCLC-9*	Full set	9	9,230,514	1.26E−05	0.0325983	5.1	*qLTSS9-1*	Schläppi et al., 2017
*qPLR-9-3*	PLR	*qGI-9-2*	Full set	9	15,399,656	4.05E−05	0.0420114	8.6		
*qPLR-9-4*	PLR	*qGI-9-3*	Full set	9	16,325,535	4.05E−05	0.0420114	8.6	*qSWTCT9; qCTS9-8*	Wang et al., 2016; Shakiba et al., 2017
*qLTG-10*	LTG	*qGI-10; qPLR-10*	Full set	10	13,897,640	6.19E−09	1.60E-05	4.2		
*qGI-10*	GI	*qLTG-10; qPLR-10*	Full set	10	13,897,640	1.21E−06	0.0062886	1.3		
*qLTG-11-2*	LTG		Full set	11	87,88,201	4.87E−06	0.0084191	10.6		

a LTG, low-temperature germinability; NTG, normal-temperature germinability; GI, germination index; CLC, coleoptile length after cold exposure; PLR, plumule growth after cold exposure; PLRR, plumule growth rate after cold exposure.

Considering the reoccurring SNPs and very closely linked SNPs in multiple chilling indices of the 51 QTLs, 31 unique QTL regions were identified to be significantly associated with cold tolerance indices within well-fitted QQ plots. Out of these 31 regions, 17 of them harbored at least 2 QTLs (QTL clusters) from the various chilling indices. For example, five QTLs identified from the five cold indices (*qLTG-1-1*, *qGI-1-1*, *qCLC-1-2*, *qPLR-1*, and *qPLRR-1*) shared the same SNP peak marker at position 29.9 Mb on chromosome 1; four QTLs from the four indices (*qLTG-5-2*, *qCLC5*, *qGI5-2*, and *qPLR5*) share the same SNP peak at 7.2 Mb on chromosome 5; four other QTLs also shared with the same SNP peak at 14.6 Mb, where 3 QTLs were from all accessions (*qGI-9-1*, *qPLR-9-2*, *qPLRR-9*) and the other one was from the *indica* subspecies (*qInPLR-9*); another four QTLs shared a very closely linked region between 17.7–117.8 Mb on chromosome 3, where two of them were from all panel (*qLTG-3-1* and *qGI-3-1*) and the other two were from the *japonica* group (*qJaLTG-3* and *qJaGI-3*); a QTL from the whole set (*qPLRR-6-1*) shared a similar region with two other QTLs from *japonica* (*qJaLTG-6-2* and *qJaGI-6-3*) at position 17.1–17.8 Mb, three QTLs (*qLTG-10*, *qGI-10*, and *qPLR-10*) shared the same SNP peak at 13.9 Mb on chromosome 10; while another three QTLs also shared the same SNP peak at 24.8 Mb where 2 of them were from the whole set (*qCLC-11-1* and *qPLR-11*) and the other one was from the *indica* group (*qInPLR11*); the rest of the QTL clusters consisted of two QTLs located on chromosomes 1, 2, 3, 4, 5, 9, 11, and 12.

### GWAS of Chilling Tolerance Indices for *japonica*

At cut-off p-value of < 0.001, we identified 20 QTLs associated with the chilling tolerance indices in *japonica* subspecies, 2 of the SNPs were detected at FDR < 0.1 (**Supplementary Table 2**; **Supplementary Figure 2**). Among the 20 QTLs, we identified 9, 8, and 3 QTLs associated with LTG, GI, and CLC, respectively. The phenotypic variance explained by the QTLs were in the range of 12.91% to 21.07% for LTG, 12.61% to 20.32% for GI, and 15.79% to 20.54% for CLC. Considering the reoccurring SNPs in multiple chilling indices and linked SNPs, we identified 12 unique QTL regions. Among these 12 QTL regions, only 3 of them contained a single QTL; while the rest harbored at least 2 QTLs. For example, three of the QTL regions were shared by QTLs from the whole panel, two regions were on chromosomes 3 and 6 as mentioned above and the other region was at position 9.2 Mb on chromosome 9, which shared by 2 QTLs (*qCLC-9* and *qJaCLC-9*). A QTL from the *japonica* group (*qJaCLC-2*) also shared a similar region at 0.6 Mb to 0.9 Mb on chromosome 2 with a QTL from the *indica (qInPLR-2*); the rest of the QTL regions contained two QTLs each identified on chromosomes 1, 5, 6, 8, and 12.

### GWAS of Chilling Tolerance Indices for *indica*

At cut-off p-value of < 0.001, we identified 9 QTLs associated with the chilling tolerance indices in *indica* subspecies. Among the nine QTLs, two QTLs each were found to be associated with LTG, GI, CLC, and three QTLs were with PLR (**Supplementary Table 2**; **Supplementary Figure 3**). Considering the reoccurring SNPs or closely linked SNPs, we identified seven unique QTL regions in *indica* subspecies. Among these regions, 4 of them harbored more than one QTL: a QTL on chromosome 9 (*qInPLR-9*) shared the same SNP peak at position of 14.6 Mb with three other QTLs detected from all accessions as mentioned above; another QTL on chromosome 11 (*qInPLR-11*) shared a SNP peak at position 24.8 Mb with two other QTLs from the whole set (*qCLC-11-1* and *qPLR-11*); q*InCLC-11* shared a SNP peak at 25.6 Mb with *qCLC-11-2* on chromosome 11; and *qIn-PLR-2* shared a closely linked SNP peaks on chromosome 2 as mentioned above; a SNP peak at 1.4 Mb on chromosome 6 was shared by *qInLTG-6* and *qInGI-6*; another peak SNP at position 20.8 Mb on chromosome 7 was shared by *qInLTG-7* and *qInGI-7.* The phenotypic variance explained by the significant SNPs were in the range of 9.96% to 11.38% for LTG, 9.78% to 12.03% for GI and 12.51% to 16.2% for CLC, and 8.85% to 10.75% for PLR, respectively.

### Candidate Gene and QTL Comparisons

Among the 31 unique QTL regions (*p* < 0.001) associated with chilling indices of the whole set of accessions, we identify 10 loci potentially co-localized with the previously identified genes/QTLs related to cold stress in rice, including cold tolerance during germination, seedling and reproductive stage, and cold recovery (**Table 4**; **Supplementary Table 2**).

A QTL associated with GI located on chromosome 5, *qGI-5-3*, was identified to be positioned at 127.9 kb away from the *OsRAN2* gene (Os05g0574500) previously reported to be responsible for cell division in cold condition (Chen et al., 2011). Another QTL for GI, *qGI-7* was found to be 141.6 kb away of *Omega-3 fatty acid desaturase* (Os07g0693800) and *qCTS7-5* which were reported to be responsible for cold tolerance at seedling stage (Wang et al., 2016). A few QTLs on chromosome 9 at around significant peak at 14.6 Mb which were significantly associated with GI, PLR (whole panel and *indica*), and PLRR, located in the vicinity of *WRKY transcription factor* (Os09g0417600) previously reported to cause increasing tolerance to cold stress in rice (Yokotani et al., 2013) and *qPGCG9-2* which was previously reported as a QTL controlling plumule growth recovery rate under cold stress during seedling stage (Schläppi et al., 2017), and 117 kb away from *OsWRKY76*, a gene similar to BRI1-KD interacting protein 120 (Os09g0410300) related to cold tolerance and *qCTS-9* previously reported related to tolerance during seedling stage (Peng et al., 2010). A SNP peak at 9.2 Mb on chromosome 9 for CLC detected by the whole set of accessions and the *japonica* panel, *qCLC-9* and *qJaCLC-9* were positioned at a distance of 169.5 kb away from *qLTSS9-1*, a QTL responsible for cold tolerance at seedling stage (Schläppi et al., 2017). A QTL associated with PLR, *qPLR-9-1* found to be 21.39 kb away from *qCTS9-2* discovered to be associated with seedling growth under cold stress (Wang et al., 2016). A SNP peak at position 24.9 on chromosome 11 associated with CLC and PLR of the full set and PLR of the *indica* was potentially colocalized with *qCTS11-9* previously reported to be responsible for cold tolerance during seedling growth (Wang et al., 2016). Similarly, another SNP peak at position 25.1 Mb on chromosome 11 associated with CLC of the full set and *indica* was 159 kb away from *qCTS11-10*, a QTL responsible for cold tolerance in seedling stage (Wang et al., 2016).

Several of our reported QTLs are found to be located in close vicinity of previously reported QTLs controlling for cold tolerance at reproductive stage in rice. For examples, a SNP peak at position 26.2 Mb on chromosome 2 associated with both PLR and PLRR was found at a distance of 109.7 kb away from the previously reported QTLs *qSWTPNCT2-2* and *qnob-5* (Shakiba et al., 2017); *qCLC-7* was identified at 135.7 kb away from *qSWTCT7* (Shakiba et al., 2017); a SNP peak at 16.3 Mb on chromosome 9 associated with GI and PLR was 214.46 kb away from a QTL for cold tolerance at reproductive stage *qSWTCT9* (Shakiba et al., 2017) and 143.79 kb away from *qCTS9-8*, a previously identified QTL for cold tolerance at seedling stage (Wang et al., 2016).

Among the 13 unique QTL regions in *japonica*, we found four GWAS sites potentially colocalized with previously identified QTLs/genes. A QTL, *qJaCLC-1-1* associated with CLC in *japonica* subspecies was found to be 140 kb distance away from *qCTS1-5*, a QTL previously reported to be responsible for cold stress tolerance in the seedling stage in rice (Wang et al., 2016) and 242.2 kb away from *qCTGERM1-8*, a QTL controlling cold stress tolerance in germination stage (Shakiba et al., 2017). Another QTL, *qJaGI-6-1* was at distance of 196.93 kb away from a QTL, *qCTS6-2* previously reported to be responsible for cold tolerance at seedling stage (Wang et al., 2016). A QTL for CLC, *qJaCLC-9* was found to be potentially collocated with a QTL, *qLTSS9-1* previously reported to be controlling for cold tolerance during seedling stage in rice (Schläppi et al., 2017). A QTL on chromosome 9, *qJaLTG-11* was identified to be potentially collocated with a QTL, *qCTGERM11-1*, previously reported to be controlling for cold tolerance during germination stage (Shakiba et al., 2017).

Among the seven unique QTL regions identified in *indica* subspecies, 6 of them were found to be potentially colocalized with previously identified genes/QTLs. A significant SNP on chromosome 6 at position 1.4 Mb was associated with LTG and GI in our study was only 10.51 kb away from *OsDREB1C* (Os06g0127100), which was reported to be associated with cold, drought and stress tolerance in rice (Ito et al., 2006). The SNP peak at 20.8 Mb on chromosome 7 which shared by *qInLTG-7* and *qInGI-7* was potentially collocated with *OsFAD9*, *FAD8* (Os07g0693800), and *qCTS7-*5, which were previously reported to be controlling for cold tolerance in the seedling stage in rice (Wang et al., 2016). A QTL, *qInCLC-8* on chromosome 8 at position 10.36 Mb was found to be potentially colocalized with *qCTGERM8-1*, and *qCTS8-2*, which were responsible for cold tolerance during germination and seedling stage in rice (Wang et al., 2016; Shakiba et al., 2017). The QTL regions on chromosome 9 and 11 that were also shared with the cold indices of the whole set have been discussed above.

## Discussion

It has been a challenge to map loci associated with abiotic stress tolerance traits like cold tolerance due to the polygenic nature of the loci (Shakiba et al., 2017). The separate GWAS analysis of low-temperature germinability (LTG) and germination index (GI) helped us to discover whether the chilling tolerance was due to the inherent cold tolerance ability or due to high seedling vigor. Moreover, the plumule length traits (PLR and PLRR assays) helped us to determine if there is a quantitative effect on subsequent growth and development of seedlings after a recovery period at normal temperature. These assays are important to measure, as some accessions with good LTG indices did not grow well after a temperature shift to 30°C and *vice versa*. All of these assays may address a realistic scenario in direct-seeding method of rice cultivation where germinating rice seeds or young seedlings may get exposed to warm-growth promoting temperature after an extended period of cold exposure.

The inbred lines developed at the Beaumont Research Center that were used in our study generally had a good level of tolerance under cold stress during germination, including the recovery phase. On the other hand, the *aus* sub-population had the lowest value of CLC while *O. glaberrima* species had the lowest values of GI, PLR, and PLRR demonstrating that these groups are not good sources of cold-tolerant genes. However, our sample size representing *O. glaberrima* might be too small; therefore, research focusing on this species with a greater number of samples is needed to have more conclusive results. Accessions belonging to the NERICA lines are found to have good GI, CLC, PLR, and PLRR. The *aromatic* and *aus* groups were found to have low tolerance to cold stress indicated by the low values of different chilling indices which were similar to the findings of other studies (Schläppi et al., 2017; Shakiba et al., 2017).

Interestingly, we didn't find a significant difference in LTG between highly tolerant *temperate japonica* and *indica* groups. As the LTG values observed were relatively similar between different sub-populations, there is a chance that both *indica* and *japonica* subspecies may carry the alleles contributing to superior LTG abilities. This also shows that there are many accessions of *indica* species which have good germination under cold stress. This is in agreement with the recent findings of Shakiba et al. (2017) where they had identified *indica* specific LTG QTL and have reported that both *indica* and *japonica* sub-species are expected to have alleles contributing to superior LTG abilities. On the contrary, we observed significantly lower values of CLC, PLR, and PLRR of the *indica* group than the *temperate japonica*. These findings showed that although the *indica* group has good germination ability under cold stress conditions, their growth rapidly gets retarded under cold condition.

The results of our study showed that the Texas breeding lines, *temperate japonica* and *tropical japonica*, were more tolerant of cold stress whereas *aus*, *aromatic*, and *indica* lines were more susceptible to cold conditions. The phenotypic measurement of different chilling indices revealed that *japonica* subspecies were generally more tolerant than *indica*. This finding is consistent with previous findings (Cui et al., 2002; Morsy et al., 2005; Lv et al., 2016). This could be because in general *indica* accessions are more adapted to higher temperature regions of low latitude while *japonica* accessions are more adapted to lower temperature regions of a higher latitude and higher elevations. This history of adaptation between *indica* and *japonica* accessions is also reflected by genes having a ratio of nonsynonymous vs synonymous substitution rates (Ka/Ks ratio) greater than 1.0, which indicates positive selection, as shown by a study between the *indica* rice 9311 and Nipponbare (Sun et al., 2015). A comparison of the QTLs in our study having FDR < 0.05 with the list of 3,340 genes with Ks of zero and Ka above zero (**Table S1** from Sun et al., 2015) revealed 5 out of 13 cold-tolerance loci in our study contain genes under selection between *indica*/*japonica* within 250 kb, including a match within 7 kb of our QTL cluster at 29.9 Mb on chromosome 1 (data not shown). Although this may be suggestive that *indica*/*japonica* alleles at some cold tolerance loci may have been under selection, further analysis would be needed to validate these results. In any case, the presence of differences in the genetic architecture of cold tolerance among different subspecies and sub-populations analyzed in this study provides opportunities for enhancing cold tolerance through molecular breeding.

Spearman's correlation analysis showed that all the indices were highly correlated with each other. Likewise, the heritability values of all the traits were also high. Schlappi et al. (2017) had reported that low-temperature germination (LTG) and plumule growth recovery rate (PLRR) were not correlated or weakly correlated with other indices while PLR was highly correlated with other indices. In our study, however, we found a high correlation between LTG with all other measured indices, albeit with different levels. Partly, this could be attributed to the differences in tolerance ability of the different accessions used in both studies. We also observed some significant loci detected in either *japonica* or *indica* were also observed in the whole set. This indicates that the significant SNPs detected in the whole set might come from that particular subspecies.

We observed seven SNP peaks/QTL regions that were shared between LTG and GI (**Table 4**; **Supplementary Table 2**). This is in agreement with the correlation analysis where a highly positive correlation was observed between LTG and GI (0.97), since GI is largely derived from LTG, especially for lines with similar levels of germination under normal conditions. This result also shows that the significant associations discovered from GI are mostly due to the tolerance of the accessions to cold germination and not due to the seedling vigor. There were three SNP peaks/QTL regions associated with both CLC and PLR, this may indicate that there may be some similar genetic mechanism or overlapping mechanisms underlying coleoptile length growth at low temperature and plumule recovery after cold stress exposure. Three of the significant SNPs associated with PLR were found to be associated with PLRR. This is in agreement with the highly positive correlation analysis of chilling indices PLR and PLRR (0.96). This further suggests that LTG and GI, PLR and PLRR may share some common genetic mechanisms. Fine mapping and ultimately cloning of the responsible genes could be performed to confirm whether the overlapping QTLs associated with one or more genetic factors.

Some of the significant SNPs identified from our GWAS study were located within the LD regions of known cold tolerance genes or previously reported QTLs, including 10 in the whole panel, 4 in *japonica*, and 6 in *indica*. In addition to validating our GWAS results, many of the identified QTLs near the previously mapped chilling tolerance related genes in rice help us to narrow down the QTL region and provide further support of the location of the underlying genes. Among the most interesting regions identified were near those QTLs which were found to be located very close to the genes involved in cold stress tolerance, including *OsDREB1C* (10.51 kb) and *OSWRKY76* (117 kb).

In summary, our novel diversity panel has little overlap with previously studied rice diversity panels, including the RDP1/RDP2, USDA Rice Mini-Core, and the 3,000 Rice Genomes panel, which may lead to the discovery of additional novel genetic loci for cold tolerance in rice. The GWAS QTLs detected in our study may provide additional information on the genetic structure of cold tolerance and recovery during germination in rice. In the future, some selected QTLs can be targeted for further molecular studies to better understand the mechanisms underlying cold tolerance and recovery of germinating rice seeds. Some selected cold tolerance-associated SNP markers can also potentially be used for MAS in rice improvement efforts. Further, a set of new highly tolerant rice accessions can potentially be used as novel donors for further genetic studies and crop improvement programs.

## Data Availability Statement

The genotyping data has been deposited at Dryad (https://doi.org/10.5061/dryad.q83bk3jdt). 

## Author Contributions

RT and ES designed the experiment. ES conceived the project. MT, ES, and RET developed the rice panel. RET and RT performed seed multiplication and post-harvest processes. RT performed the experiment. RT and ES analyzed data and wrote the manuscript. MT and RET edited the manuscript. All read and approved the manuscript.

## Funding

This work was supported by the Texas A&M AgriLife Research and the USDA NIFA Hatch projects # 1009299 and 1009300.

## Conflict of Interest

The authors declare that the research was conducted in the absence of any commercial or financial relationships that could be construed as a potential conflict of interest.
