# Supplementary material for: Genome-Wide Association Mapping to Identify Genetic Loci for Cold Tolerance and Cold Recovery During Germination in Rice
Source: Front Genet. 2020 Feb 21;11:22. doi: 10.3389/fgene.2020.00022 (PMC7047875; doi:10.3389/fgene.2020.00022)
Supplement: Supplementary file 1 [file DataSheet_1.pdf]

(A). Low-temperature germinability (LTG)

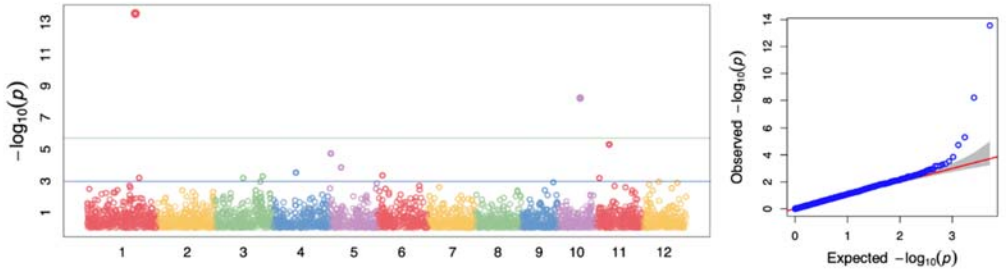

(B). Plumule length recovery rate (PLRR)

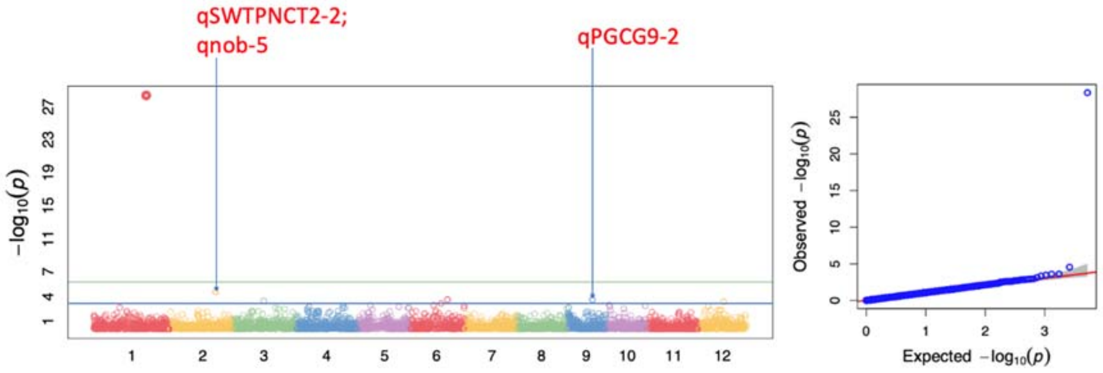

Supplementary Figure 1. Manhattan and QQ plots of the whole panel for low-temperature germinability (A), and for plumule length recovery rate (B). The solid blue line shows the p-value 0.001 significant threshold; while the solid green line shows the Bonferroni correction.
