# Supplementary material for: Genome-Wide Association Mapping to Identify Genetic Loci for Cold Tolerance and Cold Recovery During Germination in Rice
Source: Front Genet. 2020 Feb 21;11:22. doi: 10.3389/fgene.2020.00022 (PMC7047875; doi:10.3389/fgene.2020.00022)
Supplement: Supplementary file 2 [file DataSheet_2.pdf]

(A). Low-temperature germinability (LTG)

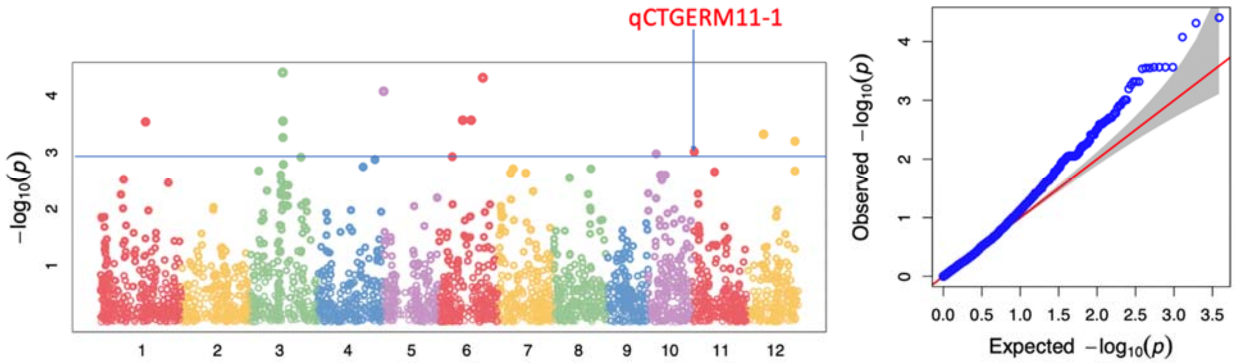

(B). Germination index (GI)

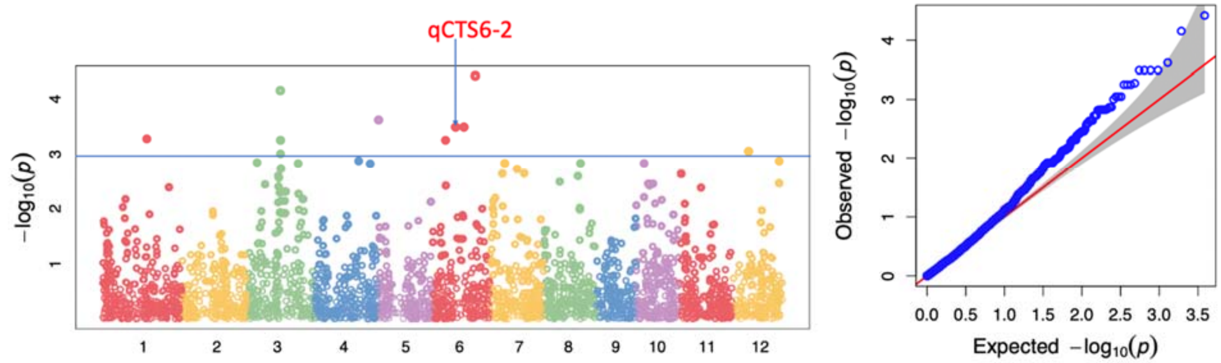

(C). Coleoptile length under cold conditions (CLC)

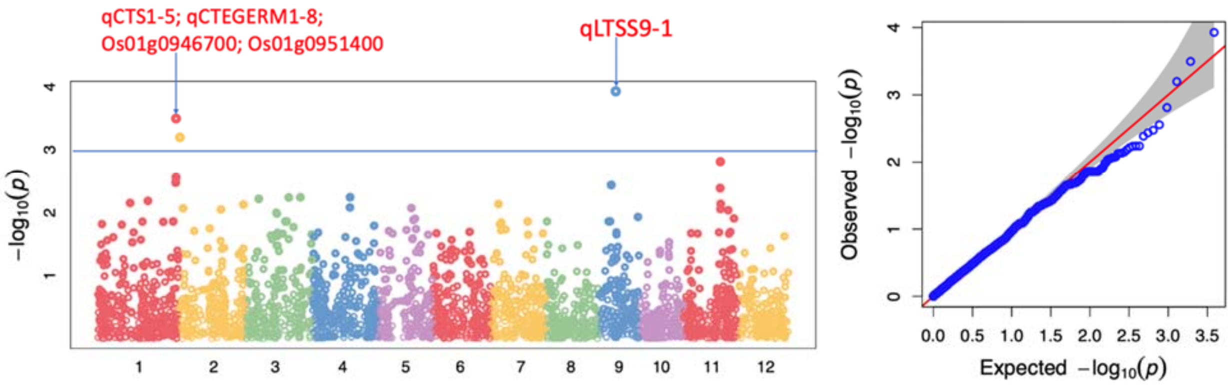

Supplementary Figure 2. Manhattan and QQ plots of the *Japonica* panel for low-temperature germinability (A), germination index (B), and coleoptile length (C). The solid blue line shows the p-value 0.001 significant threshold.
