# Supplementary material for: Genome-Wide Association Mapping to Identify Genetic Loci for Cold Tolerance and Cold Recovery During Germination in Rice
Source: Front Genet. 2020 Feb 21;11:22. doi: 10.3389/fgene.2020.00022 (PMC7047875; doi:10.3389/fgene.2020.00022)
Supplement: Supplementary file 5 [file Table_2.docx]

**Supplemental Table 2.** QTLs with FDR > 0.05 detected in the analysis of the whole panel, *Japonica* and *Indica* subsets, and colocalized genes and QTLs.

| **QTL ID** | **Trait^a^** | **Colocated QTL in this study** | **Group** | **Chr.** | **Position (bp)** | **P-value** | **FDR** | **R^2^** | **Potentially Colocated QTL/ gene** | **Reference** |
| --- | --- | --- | --- | --- | --- | --- | --- | --- | --- | --- |
| *qCLC-1-1* | CLC |  | Full set | 1 | 17394761 | 0.0001 | 0.1759163 | 2.4 |  |  |
| *qJaCLC-1* | CLC |  | Japonica | 1 | 41999235 | 0.00032 | 0.6148703 | 17.7 | *qCTS1-5; qCTEGERM1-8;Os01g0946700; Os01g0951400* | Wang et al., 2016; Shakiba et al., 2017 |
| *qJaLTG-1* | LTG | *qJaGI-1* | Japonica | 1 | 24115700 | 0.00029 | 0.1121089 | 15. 9 |  |  |
| *qLTG-1-2* | LTG | *qGI-1-2* | Full set | 1 | 32323875 | 0.00065 | 0.3147643 | 3.7 |  |  |
| *qGI-1-2* | GI | *qLTG-1-2* | Full set | 1 | 32323875 | 0.0002 | 0.1497895 | 3.9 |  |  |
| *qJaGI-1* | GI | *qJaLTG-1* | Japonica | 1 | 24115700 | 0.00053 | 0.1972021 | 13.8 |  |  |
| *qJaCLC-2* | CLC |  | Japonica | 2 | 855183 | 0.00063 | 0.8155076 | 15.8 |  |  |
| *qInPLR-2* | PLR |  | Indica | 2 | 568585 | 0.00072 | 0.8636363 | 9.4 |  |  |
| *qPLRR-2* | PLRR | *qPLR-2* | Full set | 2 | 26231409 | 2.85E-05 | 0.0739647 | 2.0 | *qSWTPNCT2-2; qnob-5* | Shakiba et al., 2017 |
| *qJaLTG-3* | LTG | *qJaGI-3; qLTG-3-1* | Japonica | 3 | 17803740 | 3.93E-05 | 0.0936803 | 21.1 |  |  |
| *qJaLTG-3* | LTG | *qJaGI-3* | Japonica | 3 | 17884540 | 0.00028 | 0.1121089 | 16.0 |  |  |
| *qJaLTG-3* | LTG | *qJaGI-3* | Japonica | 3 | 17950141 | 0.00028 | 0.1121089 | 16.0 |  |  |
| *qJaLTG-3* | LTG | *qJaGI-3* | Japonica | 3 | 17931536 | 0.00055 | 0.1511492 | 14.3 |  |  |
| *qLTG-3-2* | LTG | *qGI-3-3* | Full set | 3 | 29880015 | 0.00051 | 0.3077483 | 2.7 |  |  |
| *qLTG-3-1* | LTG | *qJaLTG-3; qJaGI-3* | Full set | 3 | 17741591 | 0.00066 | 0.3147643 | 0.5 |  |  |
| *qJaGI-3* | GI | *qJaLTG-3, qLTG-3-1* | Japonica | 3 | 17803740 | 6.99E-05 | 0.1347189 | 18.8 |  |  |
| *qJaGI-3* | GI | *qJaLTG-3* | Japonica | 3 | 17884540 | 0.00056 | 0.1972021 | 13.7 |  |  |
| *qJaGI-3* | GI | *qJaLTG-3* | Japonica | 3 | 17950141 | 0.00056 | 0.1972021 | 13.7 |  |  |
| *qJaGI-3* | GI | *qJaLTG-3* | Japonica | 3 | 17931536 | 0.001 | 0.2437403 | 12.4 |  |  |
| *qGI-3-2* | GI |  | Full set | 3 | 25530777 | 0.00095 | 0.3077483 | 2.5 |  |  |
| *qGI-3-3* | GI | *qLTG-3-2* | Full set | 3 | 29880015 | 0.00091 | 0.3077483 | 2.3 |  |  |
| *qGI-3-1* | GI |  | Full set | 3 | 17847337 | 0.00037 | 0.3811784 | 3.9 |  |  |
| *qCLC-4* | CLC |  | Full set | 4 | 11986644 | 0.00085 | 0.4964069 | 2.8 |  |  |
| *qLTG-4* | LTG | *qGI-4* | Full set | 4 | 14132556 | 0.0003 | 0.2586155 | 2.8 |  |  |
| *qGI-4* | GI | *qLTG-4* | Full set | 4 | 14132556 | 0.00092 | 0.3077483 | 1.8 |  |  |
| *qCLC-5* | CLC | *qLTG-5-2; qGI-5-2; qPLR-5* | Full set | 5 | 7195992 | 0.00023 | 0.294919 | 2.9 |  |  |
| *qJaLTG-5* | LTG | *qJaGI-5* | Japonica | 5 | 277001 | 8.42E-05 | 0.1081502 | 19.1 |  |  |
| *qLTG-5-2* | LTG | *qGI-5-2; qCLC-5; qPLR-5* | Full set | 5 | 7195992 | 0.00014 | 0.1463107 | 3.2 |  |  |
| *qGI-5-1* | GI | *qLTG-5-1* | Full set | 5 | 805425 | 0.00027 | 0.1595636 | 3.4 |  |  |
| *qGI-5-2* | GI | *qLTG-5-2; qCLC-5; qPLR-5* | Full set | 5 | 7195992 | 0.00028 | 0.1595636 | 2.6 |  |  |
| *qJaGI-5* | GI | *qJaLTG-5* | Japonica | 5 | 277001 | 0.00024 | 0.1779998 | 15.7 |  |  |
| *qJaLTG-6-3* | LTG | *qJaGI-6-4* | Japonica | 6 | 23319102 | 4.86E-05 | 0.0936803 | 20.5 |  |  |
| *qJaLTG-6-1* | LTG | *qJaGI-6-2* | Japonica | 6 | 12537890 | 0.00027 | 0.1121089 | 16.1 |  |  |
| *qJaLTG-6-1* | LTG | *qJaGI-6-2* | Japonica | 6 | 12752685 | 0.00027 | 0.1121089 | 16.1 |  |  |
| *qJaLTG-6-2* | LTG | *qJaGI-6-3* | Japonica | 6 | 17118875 | 0.00027 | 0.1121089 | 16.1 |  |  |
| *qJaLTG-6-2* | LTG | *qJaGI-6-3* | Japonica | 6 | 17174236 | 0.00027 | 0.1121089 | 16.1 |  |  |
| *qLTG-6* | LTG |  | Full set | 6 | 3245705 | 0.00045 | 0.3147643 | 2.6 |  |  |
| *qInLTG-6* | LTG | *qInGI-6* | Indica | 6 | 1444280 | 0.00081 | 0.7928021 | 10.0 | *Os06g0127100* | Ito et al., 2006 |
| *qGI-6* | GI |  | Full set | 6 | 26095829 | 0.00014 | 0.1175295 | 2.9 |  |  |
| *qJaGI-6-4* | GI | *qJaLTG-6-3* | Japonica | 6 | 23319102 | 3.80E-05 | 0.1347189 | 20.3 |  |  |
| *qJaGI-6-2* | GI | *qJaLTG-6-1* | Japonica | 6 | 12537890 | 0.00032 | 0.1779998 | 15.0 |  |  |
| *qJaGI-6-2* | GI | *qJaLTG-6-1* | Japonica | 6 | 12752685 | 0.00032 | 0.1779998 | 15.0 |  |  |
| *qJaGI-6-3* | GI | *qJaLTG-6-2* | Japonica | 6 | 17118875 | 0.00032 | 0.1779998 | 15.0 |  |  |
| *qJaGI-6-3* | GI | *qJaLTG-6-2* | Japonica | 6 | 17174236 | 0.00032 | 0.1779998 | 15.0 |  |  |
| *qJaGI-6-1* | GI |  | Japonica | 6 | 7133939 | 0.00056 | 0.1972021 | 13.7 | *qCTS6-2* | Wang et al., 2016 |
| *qInGI-6* | GI | *qInLTG-6* | Indica | 6 | 1444280 | 0.00082 | 0.7990059 | 9.8 | *Os06g0127100* | Ito et al., 2006 |
| *qPLR-6* | PLR |  | Full set | 6 | 14504992 | 0.00068 | 0.3518372 | 1.2 | *qFERCT6-3; Os06g0358800* | Shakiba et al., 2017 |
| *qPLRR-6-2* | PLRR |  | Full set | 6 | 21415912 | 0.00026 | 0.337968 | 0.9 |  |  |
| *qPLRR-6-1* | PLRR |  | Full set | 6 | 17750942 | 0.00076 | 0.5649619 | 2.3 |  |  |
| *qCLC-7* | CLC |  | Full set | 7 | 23425700 | 0.00096 | 0.4964069 | 1.8 | *qSWTCT7; Os07g0573800* | Shakiba et al., 2017 |
| *qInLTG-7* | LTG | *qInGI-7* | Indica | 7 | 20790840 | 0.0006 | 0.7928021 | 10.48 | *qCTS7-5; OsFAD8; Os07g0693800* | Wang et al., 2016 |
| *qInLTG-7* | LTG | *qInGI-7* | Indica | 7 | 20875408 | 0.00036 | 0.7928021 | 11.4 | *qCTS7-5; OsFAD8; Os07g0693800* | Wang et al., 2016 |
| *qGI-7* | GI |  | Full set | 7 | 29079097 | 0.00085 | 0.3077483 | 3.5 | *qCTS7-5; Os07g0693800* | Wang et al., 2016 |
| *qInGI-7* | GI | *qInLTG-7* | Indica | 7 | 20875408 | 0.00023 | 0.664814 | 12.0 | *qCTS7-5; OsFAD8; Os07g0693800* | Wang et al., 2016 |
| *qInGI-7* | GI | *qInLTG-7* | Indica | 7 | 20790840 | 0.00057 | 0.7990059 | 10.4 | *qCTS7-5; OsFAD8; Os07g0693800* | Wang et al., 2016 |
| *qInCLC-8* | CLC |  | Indica | 8 | 10358484 | 0.00049 | 0.4810555 | 12.5 | *qCTGERM8-1; qCTS8-2; Os08g0272200* | 79.516 ; 148.37 |
| *qPLR-8* | PLR |  | Full set | 8 | 17231656 | 0.0011 | 0.4749808 | 2.01 |  |  |
| *qJaCLC-9* | CLC | *qCLC-9* | Japonica | 9 | 9230514 | 0.00012 | 0.4553587 | 20.6 | *qLTSS9-1* | Schläppi et al., 2017 |
| *qGI-9-1* | GI | *qPLR-9-2; qInPLR-9; qPLRR-9* | Full set | 9 | 14648157 | 0.00013 | 0.1175295 | 2.6 | *qPGCG9-2* | Schläppi et al., 2017 |
| *qGI-9-2* | GI | *qPLR-9-3* | Full set | 9 | 15399656 | 0.00034 | 0.1595636 | 2.3 |  |  |
| *qGI-9-3* | GI | *qPLR-9-4* | Full set | 9 | 16325535 | 0.00034 | 0.1595636 | 2.3 | *qSWTCT9; qCTS9-8* | Shakiba et al., 2017; Wang et al., 2016 |
| *qPLR-9-1* | PLR |  | Full set | 9 | 1727512 | 0.00042 | 0.2433587 | 0.6 | *qCTS9-2* | Wang et al., 2016 |
| *qInPLR-9* | PLR | *qGI-9-1; qPLR-9-2; qPLRR-9* | Indica | 9 | 14648157 | 0.00031 | 0.337968 | 10.8 | *qCTS-9; OsWRKY76; Os09g0410300* | Peng et al., 2010 |
| *qPLRR-9* | PLRR | *qGI-9-1; qPLR-9-2; qInPLR-9* | Full set | 9 | 14648157 | 0.00025 | 0.337968 | 2.7 | *qPGCG9-2* | Schläppi et al., 2017 |
| *qPLR-10* | PLR | *qLTG-10; qGI-10* | Full set | 10 | 13897640 | 8.62E-05 | 0.0638234 | 1.3 |  |  |
| *qInCLC-11* | CLC | *qCLC-11-2* | Indica | 11 | 25591959 | 8.40E-05 | 0.2461104 | 16.2 | *qCTS11-10* | Wang et al., 2016 |
| *qInCLC-11* | CLC |  | Indica | 11 | 26028278 | 0.00032 | 0.4694276 | 13.4 |  |  |
| *qCLC-11-1* | CLC |  | Full set | 11 | 24650416 | 0.00092 | 0.4964069 | 3.2 |  |  |
| *qCLC-11-1* | CLC | *qPLR-11; qInPLR-11* | Full set | 11 | 24782915 | 0.00057 | 0.4964069 | 2.7 | *qCTS11-9* | Wang et al., 2016 |
| *qCLC-11-2* | CLC | *qInCLC-11* | Full set | 11 | 25591959 | 0.0008 | 0.4964069 | 3.0 | *qCTS11-10* | Wang et al., 2016 |
| *qJaLTG-11* | LTG |  | Japonica | 11 | 1378959 | 0.00098 | 0.222613 | 12.9 |  |  |
| *qJaLTG-11* | LTG |  | Japonica | 11 | 1470158 | 0.00098 | 0.222613 | 12.9 | *qCTGERM11-1* | Shakiba et al., 2017 |
| *qLTG-11-1* | LTG |  | Full set | 11 | 2742952 | 0.00067 | 0.3147643 | 3.1 |  |  |
| *qPLR-11* | PLR | *qCLC-11-1; qInPLR-11* | Full set | 11 | 24782915 | 0.00023 | 0.1484168 | 1.7 | *qCTS11-9* | Wang et al., 2016 |
| *qInPLR-11* | PLR | *qCLC-11-1; qPLR-11* | Indica | 11 | 24782915 | 0.001 | 0.8636363 | 8.9 | *qCTS11-9* | Wang et al., 2016 |
| *qCLC-12* | CLC |  | Full set | 12 | 14661596 | 0.0008 | 0.49640693 | 2.9 |  |  |
| *qJaLTG-12-1* | LTG | *qJaGI-12* | Japonica | 12 | 9074224 | 0.00048 | 0.1426261 | 14.7 |  |  |
| *qJaLTG-12-1* | LTG | *qJaGI-12* | Japonica | 12 | 9129374 | 0.00048 | 0.1426261 | 14.7 |  |  |
| *qJaLTG-12-1* | LTG | *qJaGI-12* | Japonica | 12 | 9181124 | 0.00048 | 0.1426261 | 14.7 |  |  |
| *qJaLTG-12-2* | LTG |  | Japonica | 12 | 25802032 | 0.00064 | 0.1644111 | 14.0 |  |  |
| *qJaGI-12* | GI | *qJaLTG-12-1* | Japonica | 12 | 9074224 | 0.0009 | 0.2437403 | 12.6 |  |  |
| *qJaGI-12* | GI | *qJaLTG-12-1* | Japonica | 12 | 9129374 | 0.0009 | 0.2437403 | 12.6 |  |  |
| *qJaGI-12* | GI | *qJaLTG-12-1* | Japonica | 12 | 9181124 | 0.0009 | 0.2437403 | 12.6 |  |  |
| *qGI-12* | GI |  | Full set | 12 | 10469146 | 0.00062 | 0.267109 | 2.4 |  |  |
| *qPLRR-12* | PLRR |  | Full set | 12 | 14470116 | 0.00046 | 0.3986086 | 0.8 |  |  |

^a^LTG: low-temperature germinability; GI: germination index; CLC: coleoptile length after cold exposure; PLR: plumule growth after cold exposure; PLRR: plumule growth rate after cold exposure
